# Features of fibrosis regression abound in “non-cirrhotic” patients with resected hepatocellular carcinoma

**DOI:** 10.1371/journal.pone.0267474

**Published:** 2022-05-12

**Authors:** Christine E. Orr, Peter L. Wang, Lina Chen, Tao Wang

**Affiliations:** 1 Department of Pathology and Molecular Medicine, Queen’s University, Kingston, Ontario, Canada; 2 Department of Medicine, Division of Gastroenterology, Queen’s University, Kingston, Ontario, Canada; 3 Department of Laboratory Medicine and Molecular Diagnostics, Sunnybrook Health Sciences Centre, University of Toronto, Toronto, Ontario, Canada; Universita degli Studi Di Cagliari, ITALY

## Abstract

Cirrhosis is a major risk factor for developing hepatocellular carcinoma (HCC). However, many surgically resected HCCs are presumably non-cirrhotic. The dynamic nature of chronic liver disease leads to periods of hepatic repair and fibrosis regression. We hypothesize that most resected HCCs, including those from non-cirrhotic patients, exhibit features of fibrosis regression in their background liver, suggesting previously more advanced liver disease. We reviewed the histology of 37 HCC resections performed between 2005–2020, including 30 from non-cirrhotic patients. The non-neoplastic liver was evaluated for features of liver disease and of the hepatic repair complex (HRC). CD34 immunohistochemistry was performed as a marker of sinusoidal capillarization. CD34 staining was evaluated manually and also by a digital image classifier algorithm. Overall, 28 cases (76%) had a high number of fibrosis regression and hepatic repair features (≥4 out of 8 features). Amongst the 30 non-cirrhotic patients, 21 (70%) showed a high number of repair features. Relative CD34 expression was increased in cases with a high number (≥4) of HRC features versus a low number (≤3) of features (p = 0.019). High HRC cases were more likely to exhibit nodular circumferential CD34 staining (p = 0.019). Our findings suggest that most resected HCC from non-cirrhotic patients display features of fibrosis regression in their background liver. Thus many, if not most, HCC patients who are “non-cirrhotic” may in fact have regressed cirrhosis. This finding reinforces that patients with regressed cirrhosis continue to be at high risk for HCC.

## Introduction

Hepatocellular carcinoma (HCC) is the most common primary liver malignancy (75–85%) and the fourth leading cause of cancer-related deaths [1]. Cirrhosis is the major risk factor for developing HCC with 80–90% of HCC cases developing on a background of cirrhosis [2]. Cirrhosis is the final stage of fibrosis, histologically defined by bands of fibrosis forming discrete hepatocyte nodules. However, a minority of HCC patients (10–20%) are clinically labelled as non-cirrhotic and these patients are disproportionately represented in resected cases, in large part because they are often better surgical candidates [3].

**Fig 1:**
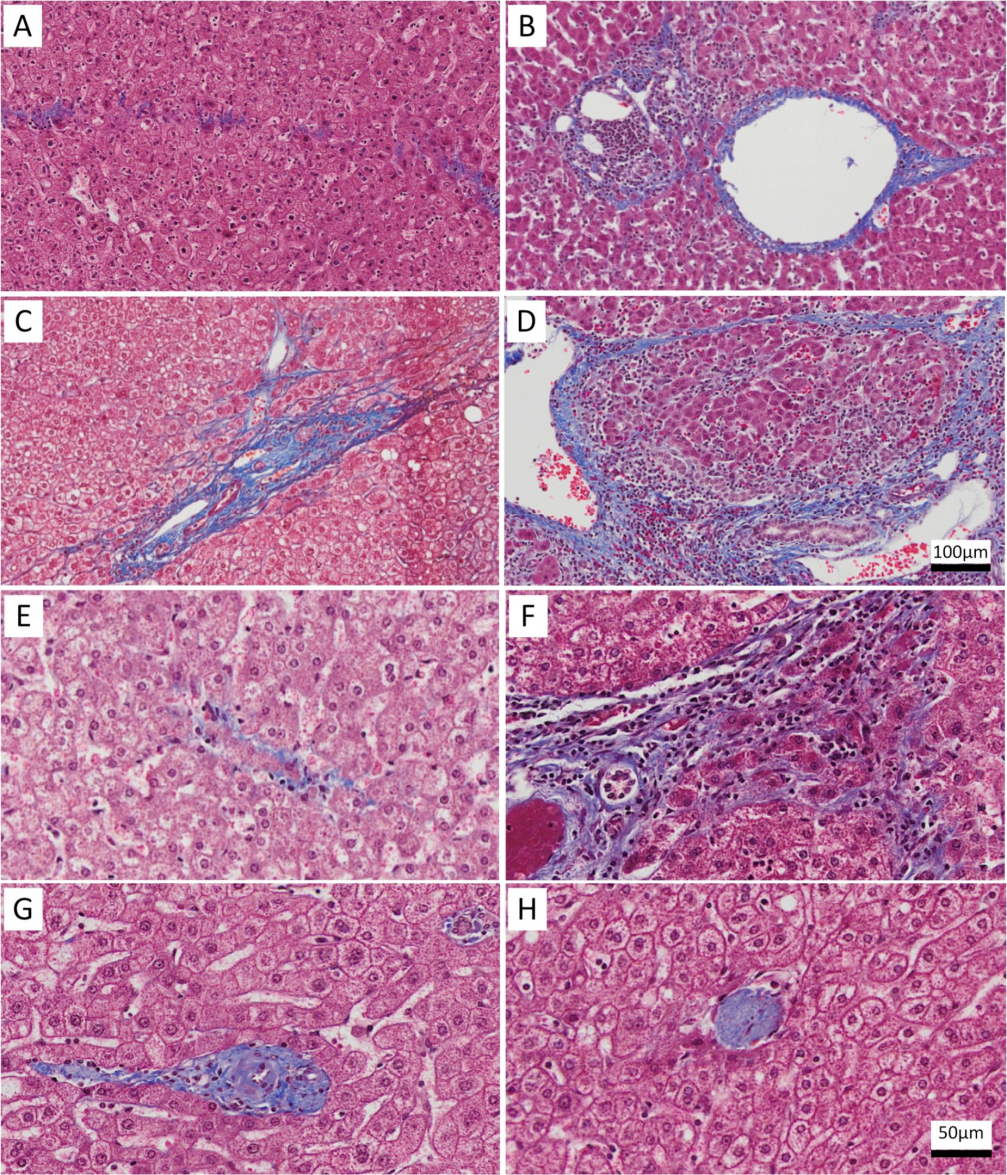
Features of the hepatic repair complex (Trichrome stain). Perforated delicate septa (A); aberrant parenchymal vein (B); delicate periportal fibrous spikes (C); minute regenerative nodule (D); hepatic vein remnant with prolapsed hepatocytes (E); hepatocytes within portal tracts or split septa (F); portal tract remnant (G); and isolated collagen bundle (H). The scale bar in panel D applies to panels A-D; the scale bar in panel H applies to panels E-H.

Regression of liver fibrosis has been demonstrated to occur in many chronic liver diseases including: hepatitis B virus (HBV), hepatitis C virus (HCV), hereditary hemochromatosis, alcoholic liver disease (ALD), non-alcoholic fatty liver disease (NAFLD), primary biliary cirrhosis, and autoimmune hepatitis [4–24]. Recent studies are conflicted regarding the relative risk of HCC in patients with histologic evidence of regression of advanced liver disease [24–27]. The histologic features of liver fibrosis regression were first described and termed the hepatic repair complex (HRC) by Wanless *et al* [4]. The eight histologic features are *perforated delicate septa*, *isolated thick collagen fibers*, *delicate periportal fibrous spikes*, *central veins remnants*, *hepatocytes within portal tracts or split septa*, *minute regenerative nodules*, *aberrant parenchymal veins*, and *portal tract remnants* (Fig 1) [4].

Progression to cirrhosis is also accompanied by a physiologic change in liver sinusoidal endothelial cells (LSECs) termed *capillarization* in which the cells lose fenestration and aberrantly express endothelial markers [28,29]. One marker that is not expressed in healthy LSECs but is expressed after capillarization is CD34 [29–31]. A study by D’Ambrosio *et al* in 2012 suggested aberrant CD34 expression was retained in HCV patients after treatment despite regression [6].

We hypothesize a significant proportion of HCC patients with surgically resected tumours, the majority of whom are clinically considered non-cirrhotic, exhibit features of repair and regression. This would imply that many non-cirrhotic patients previously had more advanced liver disease, including cirrhosis. Furthermore, we hypothesize that CD34 may be used as an adjunct marker for prior liver fibrosis and subsequent regression.

## Materials and methods

### Clinical data

The study was approved by the Queen’s University Affiliated Teaching Hospitals Research Ethics Board (Reference 6026744; initially approved 5/24/2019). Requirement for written consent was waived by the REB as this was a low-risk retrospective study where data is presented in a deidentified state. Clinicopathologic data was collected retrospectively on 37 HCC patients with liver resections performed at Kingston Health Sciences Center in Kingston, ON, Canada between 2005–2020. History of liver disease was determined based on clinical, biochemistry, serology, pathology, and imaging findings. Biochemical and serological results were retrieved from just prior to resection, and from at least two years earlier. Another 10 stage and disease matched liver biopsies were retrieved from patients without hepatocellular carcinoma and who did not have any liver disease-specific treatment to serve as histologic comparator controls.

### Histologic features

From each resection a non-neoplastic liver section was sampled from as far away from the tumour as possible. Cases where the maximum sample dimension was within 2 cm of the maximum tumour dimension were excluded from review due to tumour proximity to uninvolved liver. This section was stained with Masson’s trichrome (Sigma-Aldrich TriChrome Stain HT15-1KT and Weigert’s Iron Hematoxylin Set HT1079-1SET) and CD34 immunohistochemistry (Leica clone QBEnd/10). HCC case and control slides were randomized, assigned a study number, and reviewed by the authors (CEO and TW, both are fellowship trained GI/Liver pathologists) while blinded to the clinical history. Classification of each criteria was based on consensus opinion.

Fibrosis was staged by the Laennec system. Stage 0 is no fibrosis. Stage 1 is minimal fibrosis with no to rare thin septa, mild portal expansion, or mild sinusoidal fibrosis. Stage 2 is mild fibrosis with occasional thin septa. Stage 3 is moderate fibrosis with numerous septa and bridging up to incomplete cirrhosis. Stage 4 is cirrhosis and is subclassified into A, B, and C. Stage 4A is mild cirrhosis with nodules contoured by thin septa. Stage 4B is moderate cirrhosis with at least 2 moderately broad septa. Stage 4C is severe cirrhosis with minute nodules or very broad septa [32]. Portal inflammation was evaluated as: absent (0), mild and non-expansile (1), and moderate-severe density and/or expansile (2). Lobular inflammation was evaluated as absent (0), mild (1) with rare apoptotic hepatocytes or rare balloon cells, or moderate to severe (2) with numerous apoptotic hepatocytes or balloon cells. Steatosis was recorded as a percentage of total area involved and evaluated as: none (<5%), mild (5–33%), moderate (34–66%), and severe (>66%).

Each HRC feature (Fig 1) was screened for in the entire section and recorded as absent or present. We followed the descriptions laid out by Wanless et al [4]. Specifically, *perforated delicate septa* are discontinuous curved fibrous septa. *Isolated thick collagen fibers* are heavy collagen bundles not visibly attached to a portal structure, central vein, or septum. *Delicate periportal fibrous spikes* are remnants of periportal septa. *Hepatic vein remnants* are veins with significant or complete lumen obliteration, often associated with prolapse of hepatocytes. *Hepatocytes within portal tracts* are clusters of hepatocytes 2 or more cells in thickness located within portal tracts. *Minute regenerative nodules* are small clusters of hepatocytes admixed with ductules. *Aberrant parenchymal veins* are central veins abnormally close to portal tracts (within 5 hepatocytes). *Portal tract remnants* are portal tracts missing one of the normal structures.

### CD34 and HALO image analysis

CD34 sections were scanned into the HALO image analysis software. The image classifier algorithm was trained by manually annotating four high power fields into 3 different classes: hepatocytes, CD34+ sinusoids, and blank space. The trained classifier algorithm was run to analyze total slide areas (with exclusion of large muscular arteries and liver capsule). The algorithmic classification was visually assessed for histologic concordance as quality control. Final numerical data gave a total area of each classifier allowing for assessment of CD34 staining area to total hepatocyte area.

CD34 immunohistochemistry was also analyzed manually in a blinded manner for the presence of circumference CD34 staining, or “CD34 nodules”. CD34 nodules are considered present in a slide if 2 or more foci are identified which demonstrate nodular circumferential CD34 staining. The staining does not have to coincide with fibrous septa. The nodules must not be immediately subcapsular in location to count.

### Statistical analysis

Statistical analysis was performed using GraphPad Prism (San Diego, CA) software. Kruskal-Wallis with Dunn’s multiple comparisons correction or Mann-Whitney U test were used for comparison of CD34 image analysis relative expression results between three groups or two groups, respectively. Fisher’s exact test was used for comparison of binary outcome variables between two groups. Mann-Whitney U test was used for comparison of biochemical values between two groups. For statistical analysis, fibrosis stage 0 and 1 were grouped together, stage 2 and 3 were grouped together, and stage 4A-C were grouped together to achieve adequate specimen numbers. The number of HRC features were defined as low (0–3) vs high (4–8).

## Results

### Clinical history

A total of 37 HCC resections was collected. Surgery types included lobectomy (n = 13), segmentectomy (n = 17), or wedge resections (n = 7). Based on clinical, radiologic, and pathologic review, 32 patients had one or more etiologies of chronic liver disease. Specifically, they were HCV with ALD (n = 4), HCV alone, (n = 7), HBV (n = 4), and ALD alone (n = 8), and NAFLD (n = 9). One of the NAFLD patients had a concurrent history of alpha-1-anti-trypsin deficiency. Of the five remaining patients, three had a history of diabetes without objective (histologic or radiologic) evidence of NAFLD. Two patients did not have history of liver disease or known risk factors. Results are summarized in Table 1 by the number of HRC features.

**Table 1:** Patient demographics and number of HRC features.

Characteristic	No. of HRC features (%)		Total (%)
	0–3 (N = 9)	4–8 (N = 28)	N = 37
**Mean Age (Range)**	60 (35–76)	68 (53–84)	**66 (35–84)**
**Sex**			
Women	0	6 (21)	**6 (16)**
Men	9 (100)	22 (79)	**31 (84)**
**Chronic Liver Disease** ^a^			
HCV	1 (11)	10 (36)	**11 (30)**
HBV	1 (11)	3 (11)	**4 (11)**
Alcohol	1 (11)	11(39)	**12 (32)**
NAFLD	4 (44)	5 (18)	**9 (24)**
Alpha-1-antitrypsin	0	1 (4)	**1 (3)**
None/Unknown	2 (22)	3 (11)	**5 (14)**
**Biochemistry before HCC, Mean (Range)** ^**b**^	N = 5	N = 16	N = 21
ALT U/L	26 (22–27)	66 (13–222)	**56 (13–222)**
AST U/L	23 (17–30)	59 (24–119)	**48 (17–119)**
Bilirubin umol/L	9 (7–13)	16 (6–33)	**15 (6–33)**
Albumin g/L	43 (39–46)	36 (24–48)	**38 (24–48)**
Platelets x 10^9^/L	172 (127–245)	177 (127–289)	**176 (22–289)**

^a^Some patients have multiple causes of chronic liver disease.

^b^This represents the most recent biochemistry that is from at least 2 years before their HCC diagnosis.

Within the cohort, eighteen patients had documented treatment or recovery for their liver disease prior to HCC resection. This was in the form of antiviral medication, reduction of or abstinence from alcohol, and weight loss or diabetes treatment. Four HCV patients had prior antiviral treatment, one had Harvoni with sustained virologic response (SVR) and two had Peg-interferon/Ribavirin with SVR. The fourth patient failed Harvoni but had concurrent ALD and did abstain from alcohol. Another patient with HCV and ALD spontaneously cleared the HCV without anti-virals. The six remaining HCV patients either did not have anti-viral treatment or only received it after their resection. Of the four HBV patients, one received lamivudine prior to surgery with DNA levels becoming undetectable (6 log decrease) on subsequent follow-up. Another patient spontaneously cleared HbsAg and had undetectable HBV DNA after several decades of chronic HBV but prior to developing HCC. A third patient had low viral levels of 400 IU/mL without therapy. The last HBV patient had HbeAg negative hepatitis with a viral load of 7.8 x 10^5^ IU/mL but had not received therapy prior to surgery. Nine of twelve ALD patients (with or without concurrent HCV) reduced alcohol intake prior to HCC resection for periods ranging from 6 months to 25 years; of these five abstained completely for at least a year. Of the nine NAFLD patients, three were on diabetes medications, and one had documented weight loss of 5% (5 kg).

### Histologic features

Per Table 2, of the 37 cases, 5 did not exhibit fibrosis (stage 0) and 7 were cirrhotic (stage 4A-C). HRC features were found in 31 cases and were seen in all patients who had history of liver disease and histologic fibrosis. *Hepatocytes in portal tracts* was the most frequently observed HRC feature (29/37; 78%), followed closely by *perforated delicate septa* (76%) and *portal tract remnants* (73%). Fibrosis stage 0 cases did not exhibit any HRC features. Amongst the fibrotic cases (stages 1–4), stage did not correlate with the number of HRC features by Spearman rank correlation (p = 0.47). Of the 30 patients without cirrhosis (stage 0–3), 21 (70%) showed a high number (≥4) of HRC features. All 7 patients with cirrhosis showed high numbers of HRC features.

**Table 2:** Pathologic Features and Number of HRC Features.

Characteristic	No. of HRC features (%)		Total (%)
	0–3 (N = 9)	4–8 (N = 28)	N = 37
**Fibrosis Laennec**			
0	5 (56)	0	**5 (14)**
1	2 (22)	7 (25)	**9 (24)**
2–3	2 (22)	14 (50)	**16 (43)**
4A-C	0	7 (25)	**7 (19)**
**HRC Features**			
Hepatocytes in Portal Tracts	3 (33)	26 (93)	**29**
Perforated Delicate Septa	2 (22)	26 (93)	**28**
Portal Tract Remnants	1 (11)	26 (93)	**27**
Periportal Fibrous Spikes	0	23 (82)	**23**
Aberrant Parenchymal Veins	0	22 (79)	**22**
Isolated Thick Collagen	0	20 (71)	**20**
Minute Regenerative Nodules	0	13 (46)	**13**
Hepatic Vein Remnants	0	12 (43)	**12**
**Steatosis**			
None (<5%)	4 (44)	15 (54)	**19 (51)**
Mild (5–33%)	5 (56)	10 (36)	**15 (41)**
Moderate (34–66%)	0	3 (11)	**3 (8)**
Severe (>66%)	0	0	**0**
**Portal Inflammation**			
None	4 (44)	7 (25)	**11 (30)**
Mild	3 (33)	14 (50)	**17 (46)**
Moderate/Severe	2 (22)	7 (25)	**9 (24)**
**Lobular Inflammation**			
None	0	0	**0**
Mild	0	4 (14)	**4 (11)**
Moderate/Severe	0	0	**0**

Most cases exhibited absent to mild steatosis (34/37; 92%), and degree of steatosis did not correlate with the number of HRC features. Four cases exhibited lobular inflammation (11%), all in the context of steatohepatitis. Most cases exhibited portal inflammation (70%). The majority were mild, but 9 had moderate to severe portal inflammation and of these, 3 were HBV and 4 were HCV patients. Neither type of inflammation correlated with the number of HRC features present. Fifteen patients who had prior treatment for HCV, HBV, NAFLD, or ALD showed high numbers (≥4) of HRC features. The three NAFLD patients treated for diabetes but without specific documentation of weight loss did not have high HRC features, but they all had low stage disease (fibrosis 0–1).

### Control cases

Our 10 control biopsies included patients with chronic liver disease due to HBV (fibrosis stage 3), HCV (stages 1,3,4), NAFLD (stages 1,2,3), and ALD (stages 1,3,4) who did not have any documented disease-specific therapy. These cases spanned the etiologies and stages of our HCC cohort. On review, 5 of the 10 controls showed zero HRC features; 4 showed one feature (portal hepatocytes, minute regenerative nodule, or perforated delicate septa); 1 case showed two features (hepatic vein remnant and perforated delicate septa). Overall, none of the control cases would fall into our high HRC category.

### Biochemistry

Prior transaminases and liver function tests from at least two years before surgery were available for 21 cases (Table 1). Cases with high HRC features had higher historical levels of alanine aminotransferase (ALT) and aspartate aminotransferase (AST)(p<0.015) compared to those with low HRC features. Biochemical findings immediately prior to resection were not correlated with HRC features.

### CD34 and HALO image analysis

**Fig 2:**
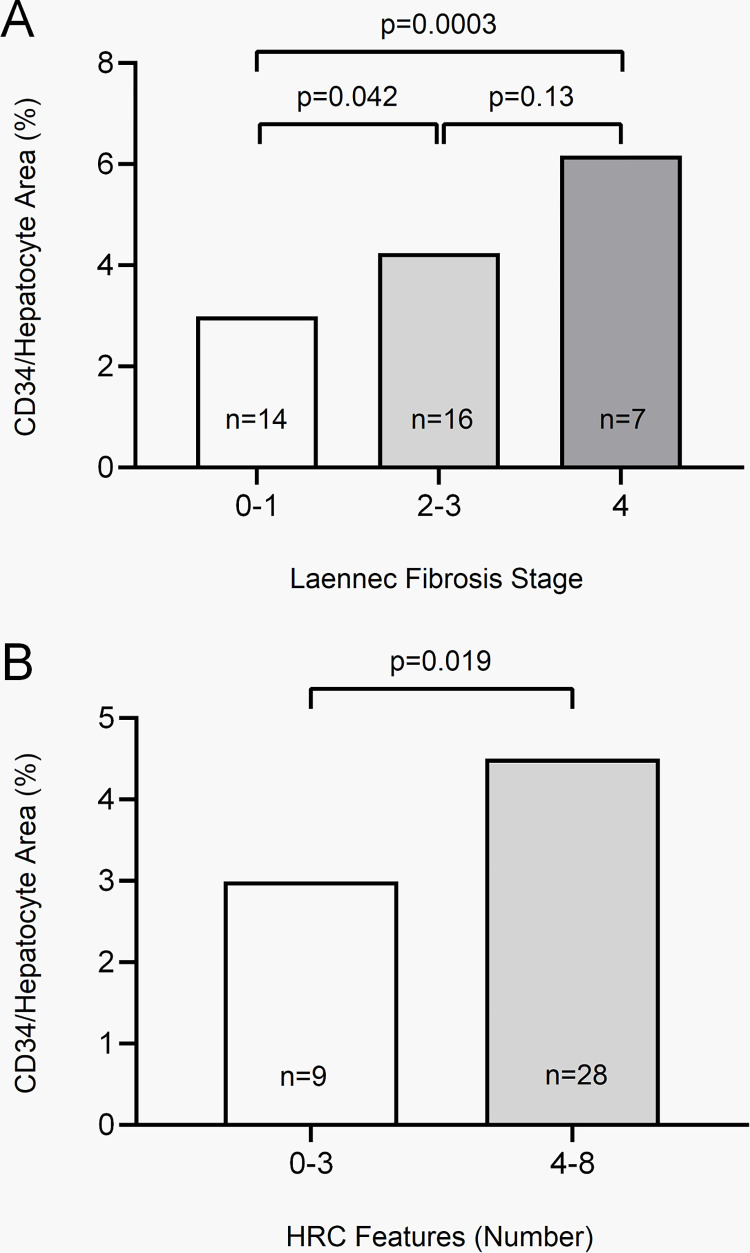
Relationship of CD34 expression to hepatocyte area by fibrosis and regression features. CD34 expression area relative to hepatocyte area based on Laennec fibrosis stage (A); CD34 expression area relative to hepatocyte area based on number of HRC features (B).

Total CD34 expression was significantly increased in cases with cirrhosis compared to cases with stage 0–1 fibrosis (p = 0.0003), and in cases with stage 2–3 fibrosis compared to cases with stage 0–1 fibrosis (p = 0.042). CD34 expression was not significantly different in cases with stage 2–3 fibrosis compared to cases with cirrhosis (p = 0.13) (Fig 2A).

**Fig 3:**
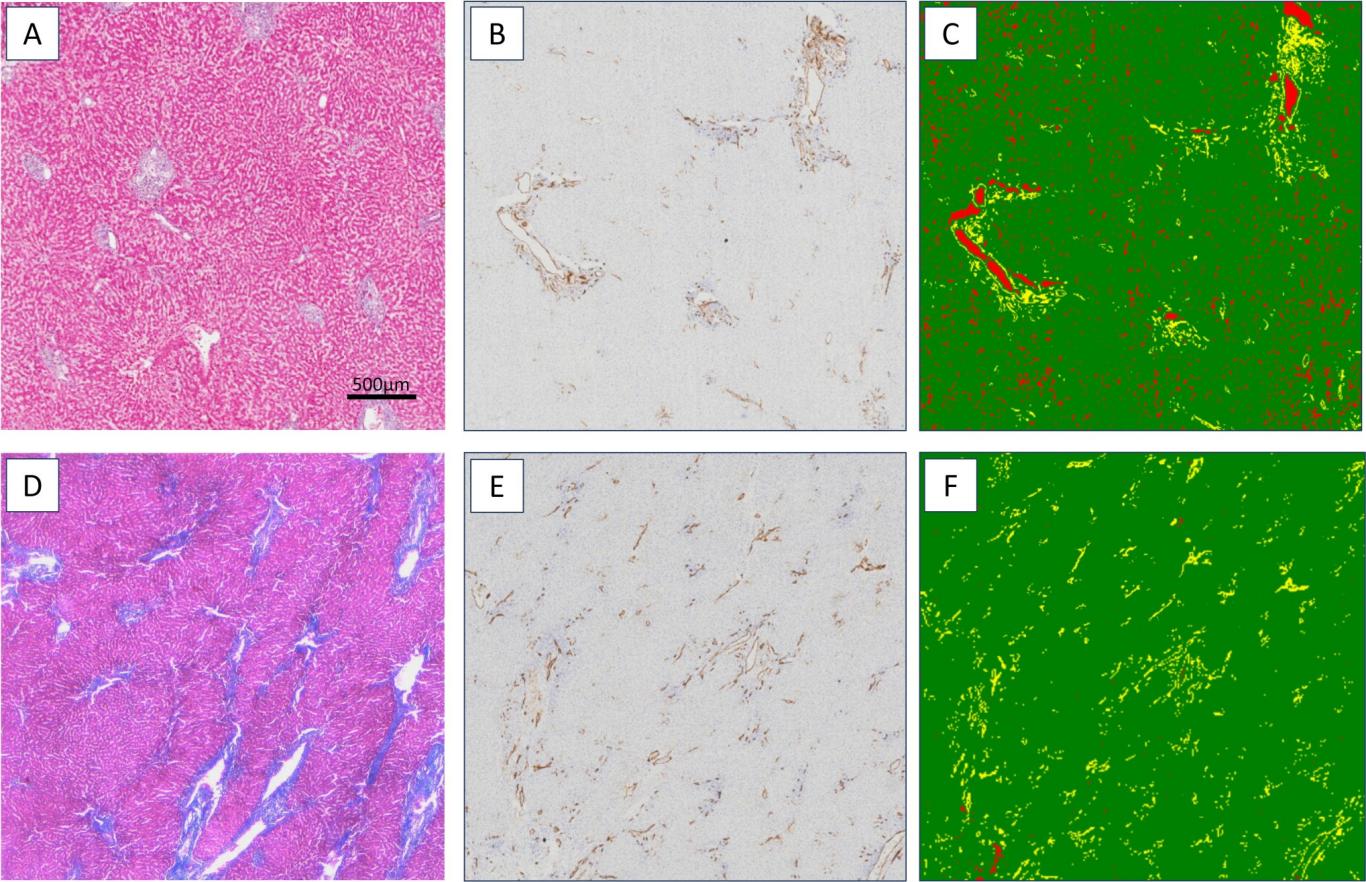
CD34 image analysis. A-C: Case with no HRC features: Trichrome stain (A); CD34 IHC (B); *HALO classifier image analysis (C). D-F: Case with 7 HRC features: Trichrome (D); CD34 IHC (E); *HALO classifier image analysis (F). *HALO classifier image analysis key: Hepatocytes (green), CD34+ sinusoids (yellow), blank space (red). Scale bar applies to all panels.

**Fig 4:**
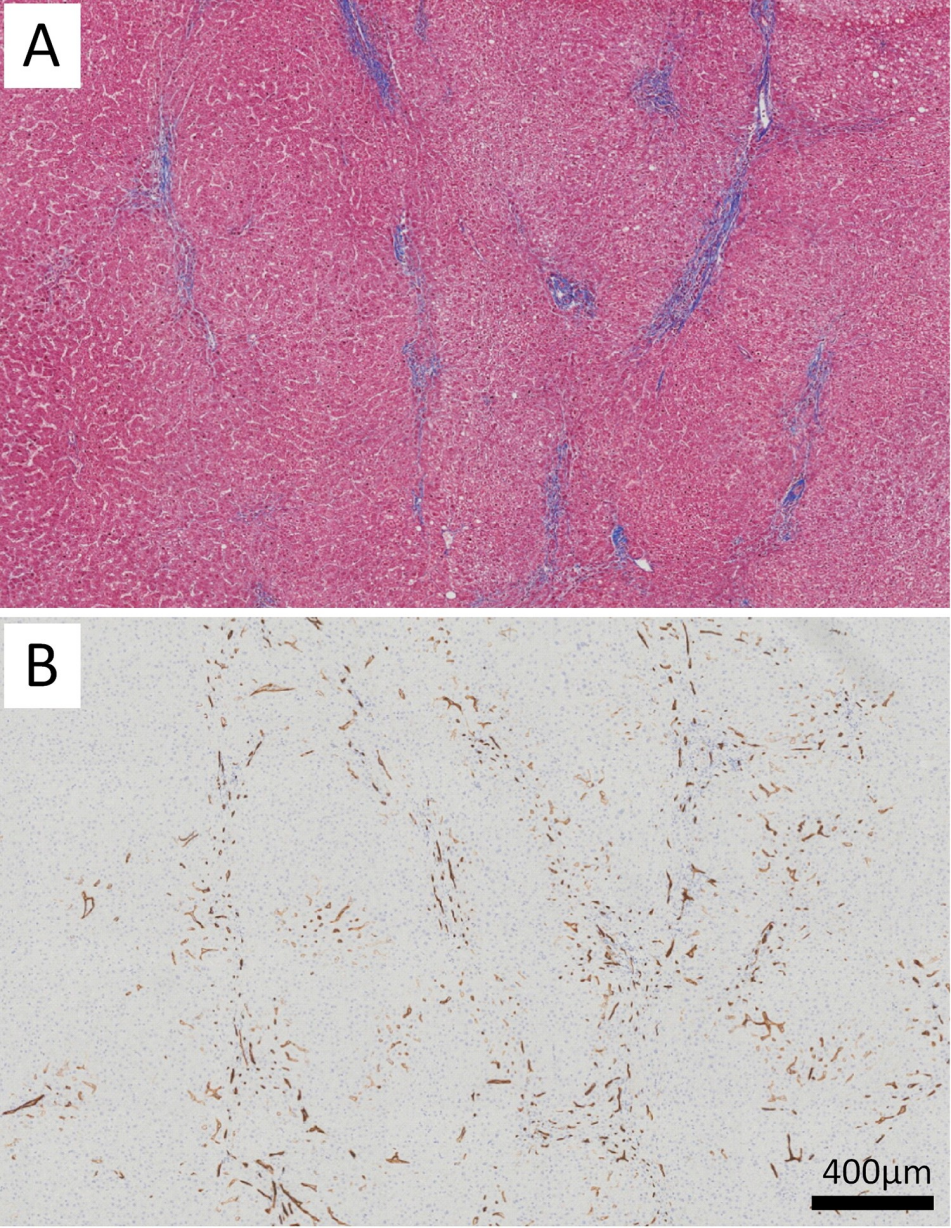
A patient with diabetes, mild steatosis, and overall stage 3 fibrosis. Trichrome stain showing an area with discontinuous septa but vague nodularity (A); CD34 IHC in the same region shows circumferential nodular staining (B). Scale bar is provided.

Total CD34 expression was also increased in cases with a high number of HRC features (4–8) compared to cases with a low number of HRC features (0–3; p = 0.019) (**Fig 2B**). The increased CD34 expression can be visually appreciated when annotated by the HALO classifier algorithm (**Fig 3**). Exhibiting ≥3.5% CD34 expression relative to hepatocyte area was associated with high numbers (≥4) of HRC features (p = 0.0078). Cases with high HRC features were more likely to exhibit circumferential nodular CD34 expression by immunohistochemistry (**Fig 4**). Only 1 of 9 cases with low HRC features exhibited CD34 nodules compared to 17 of 28 cases with high HRC features (p = 0.0072). All cirrhotic cases had circumferential CD34 nodules.

## Discussion

In our cohort, 84% of HCC resections exhibited at least some HRC features, including all cases with history of liver disease that had histologic fibrosis. More interestingly, 70% of non-cirrhotic HCC patients exhibited 4 or more features of the hepatic repair complex and around half of these also showed abnormalities in CD34 staining. Our clinical and biochemical data suggests that cases with high HRC features had higher transaminases prior to development of HCC. This supports the presence of previously active liver disease in these patients. Patients with chronic liver disease and fibrosis who underwent treatment had high numbers of repair features. These findings suggest that most HCC patients with stage 1–3 fibrosis likely previously had more advanced fibrosis or cirrhosis but underwent partial regression. Thus, the regression of cirrhosis does not completely alleviate risk of developing HCC.

A 2018 Italian study on 38 HCV patients with sustained virologic response (SVR) found no difference in HCC risk between those with histologic fibrosis regression and those without regression at 8 years of cumulative follow-up [25]. However, only six patients in the study developed HCC. The same group emphasized the lack of data on HCC risk in HCV patients with fibrosis regression due to a paucity of histological data [26]. A study employing transient elastography also showed that post-treatment HCV patients with compensated advanced liver disease still had a relatively high incidence of developing HCC (1.45 per 100 patient-years) despite lower stiffness readings [27]. Conversely, a study of 106 hereditary hemochromatosis patients discovered a significant reduction in long-term HCC risk in patients with regression [24]. The risk for HCC in patients with fibrosis regression may vary depending on the underlying etiology [33].

Part of the challenge of studying fibrosis regression is that it can be difficult to identify histologically. Regression means that fibrous septa are often partially resorbed and can mimic lower stages of fibrosis. Some features can also be patchy, make them hard to identify in small biopsies. Conversely in resections, pathologists may be more attentive to the tumour characteristics and pay less attention to subtleties in the background liver. However, our study demonstrates that these regression features can be readily identifiable in most cases of resected HCC when applying a systematic approach. *Hepatocytes within portal tracts* and *perforated delicate septa* appear to be the most frequent HRC features, and they are relatively easy to discern on trichrome stain.

Chronic liver diseases are dynamic processes and progression versus regression are not mutually exclusive. Sun *et al* argued that livers can exhibit predominantly regressive, predominantly progressive, and indeterminate patterns of disease states [34]. Our data shows this to be a reasonable stance, as 9 cases demonstrated moderate to severe portal inflammation and 4 cases showed steatohepatitis. 11 of these 13 cases showed high numbers of HRC features, suggesting that features of progressive disease and regression can occur simultaneously. Although not all patients in our HCC cohort had documented etiology-specific treatment, fluctuations in activity during their disease course, particularly amongst ALD, NAFLD/NASH, and HBV patients, may lead to dynamic shifts along a spectrum of progressive versus regressive disease.

The use of CD34 as a surrogate marker for sinusoidal capillarization correlates with both increasing fibrosis and number of HRC features. Image analysis is useful to define these differences as the area of capillarization is minute compared to hepatocyte area. Significant differences were seen between cases with mild versus severe fibrosis and in cases with low versus high numbers of HRC features. The finding of significantly higher CD34 expression in patients with fibrosis is in keeping with prior studies showing sinusoidal capillarization in cirrhotic patients [28–31]. The lack of difference in CD34 expression between cases with stage 2–3 fibrosis and cases with cirrhosis supports our hypothesis that many resected HCC patients with stage 2–3 fibrosis may be under-staged by routine histology alone due to fibrosis regression. D’Ambrosio *et al* showed retained sinusoidal capillarization in some cirrhotic HCV patients 5 years after SVR [6]. Increased CD34 expression in cases with high numbers of HRC features in our study supports these findings.

We also found that many high HRC cases, particularly those with stage 3 fibrosis, exhibited circumferential CD34 nodules that did not always coincide with existing septa. This may represent retention of abnormal CD34 expression after septal resorption. Our study suggests CD34 immunohistochemistry may be optimized to use as a supportive or screening test in equivocal or suspicious cases for fibrosis regression. Based on our findings, ≥3.5% CD34 expression relative to hepatocyte area correlates with high numbers of regression features, as does the presence of circumferential CD34 nodules.

Future investigations to address the oncogenic potential in livers with regressed fibrosis would ideally involve prospective studies with long term follow-up. Our study demonstrated that most supposedly non-cirrhotic patients with HCC exhibited multiple features of fibrosis regression. We recommend that pathologists be attentive to features of regression, as their presence may indicate more advanced physiological perturbation than current fibrosis stage alone suggests. Patients with evidence of fibrosis regression, particularly those who regressed from cirrhosis, should continue to undergo screening for neoplasia.

## Supporting information

S1 Table Clinical-pathologic parameters for study (A) and control (B) cases.
(XLSX)

S2 Table (XLSX)

## References

[1] Torbensen M, Ng I, Park N, Roncalli M, Sakamoto M. Hepatocellular Carcinoma. In: WHO Classification of Tumours: Digestive System Tumours. Lyon, France: International Agency for Research on Cancer; 2019. pp. 229–239.

[2] Fattovich G, Stroffolini T, Zagni I, Donato F. Hepatocellular carcinoma in cirrhosis: Incidence and risk factors. Gastroenterology. 2004;127: S35–50. doi: 10.1053/j.gastro.2004.09.014 15508101

[3] Delis S-G, Dervenis C. Selection criteria for liver resection in patients with hepatocellular carcinoma and chronic liver disease. World J Gastroenterol. 2008;14: 3452–3460. doi: 10.3748/wjg.14.3452 18567070 PMC2716604

[4] Wanless IR, Nakashima E, Sherman M. Regression of human cirrhosis. Morphologic features and the genesis of incomplete septal cirrhosis. Arch Pathol Lab Med. 2000;124: 1599–1607. doi: 10.5858/2000-124-1599-ROHC 11079009

[5] Laursen TL, Siggaard CB, Kazankov K, Sandahl TD, Møller HJ, Tarp B, et al. Time-dependent improvement of liver inflammation, fibrosis and metabolic liver function after successful direct-acting antiviral therapy of chronic hepatitis C. J Viral Hepat. 2020;27: 28–35. doi: 10.1111/jvh.13204 31502741

[6] D’Ambrosio R, Aghemo A, Rumi MG, Ronchi G, Donato MF, Paradis V, et al. A morphometric and immunohistochemical study to assess the benefit of a sustained virological response in hepatitis C virus patients with cirrhosis. Hepatology. 2012;56: 532–543. doi: 10.1002/hep.25606 22271347

[7] Akhtar E, Manne V, Saab S. Cirrhosis regression in hepatitis C patients with sustained virological response after antiviral therapy: a meta-analysis. Liver Int. 2015;35: 30–36. doi: 10.1111/liv.12576 24766091

[8] Manne V, Akhtar E, Saab S. Cirrhosis regression in patients with viral hepatitis B and C: a systematic review. J Clin Gastroenterol. 2014;48: e76–84. doi: 10.1097/MCG.0000000000000162 24921210

[9] Xu B, Lin L, Xu G, Zhuang Y, Guo Q, Liu Y, et al. Long-term lamivudine treatment achieves regression of advanced liver fibrosis/cirrhosis in patients with chronic hepatitis B. J Gastroenterol Hepatol. 2015;30: 372–378. doi: 10.1111/jgh.12718 25167956

[10] Tsai NC, Marcellin P, Buti M, Washington MK, Lee SS, Chan S, et al. Viral suppression and cirrhosis regression with tenofovir disoproxil fumarate in Asians with chronic hepatitis B. Dig Dis Sci. 2015;60: 260–268. doi: 10.1007/s10620-014-3336-7 25179493

[11] Marcellin P, Gane E, Buti M, Afdhal N, Sievert W, Jacobson IM, et al. Regression of cirrhosis during treatment with tenofovir disoproxil fumarate for chronic hepatitis B: a 5-year open-label follow-up study. Lancet. 2013;381: 468–475. doi: 10.1016/S0140-6736(12)61425-1 23234725

[12] Dienstag JL, Goldin RD, Heathcote EJ, Hann HWL, Woessner M, Stephenson SL, et al. Histological outcome during long-term lamivudine therapy. Gastroenterology. 2003;124: 105–117. doi: 10.1053/gast.2003.50013 12512035

[13] Chang T-T, Liaw Y-F, Wu S-S, Schiff E, Han K-H, Lai C-L, et al. Long-term entecavir therapy results in the reversal of fibrosis/cirrhosis and continued histological improvement in patients with chronic hepatitis B. Hepatology. 2010;52: 886–893. doi: 10.1002/hep.23785 20683932

[14] Hadziyannis SJ, Tassopoulos NC, Heathcote EJ, Chang T-T, Kitis G, Rizzetto M, et al. Long-term therapy with adefovir dipivoxil for HBeAg-negative chronic hepatitis B for up to 5 years. Gastroenterology. 2006;131: 1743–1751. doi: 10.1053/j.gastro.2006.09.020 17087951

[15] Papachrysos N, Hytiroglou P, Papalavrentios L, Sinakos E, Kouvelis I, Akriviadis E. Antiviral therapy leads to histological improvement of HBeAg-negative chronic hepatitis B patients. Ann Gastroenterol. 2015;28: 374–378. 26126929 PMC4480175

[16] Poynard T, McHutchison J, Manns M, Trepo C, Lindsay K, Goodman Z, et al. Impact of pegylated interferon alfa-2b and ribavirin on liver fibrosis in patients with chronic hepatitis C. Gastroenterology. 2002;122: 1303–1313. doi: 10.1053/gast.2002.33023 11984517

[17] Everson GT, Balart L, Lee SS, Reindollar RW, Shiffman ML, Minuk GY, et al. Histological benefits of virological response to peginterferon alfa-2a monotherapy in patients with hepatitis C and advanced fibrosis or compensated cirrhosis. Aliment Pharmacol Ther. 2008;27: 542–551. doi: 10.1111/j.1365-2036.2008.03620.x 18208570

[18] Serpaggi J, Carnot F, Nalpas B, Canioni D, Guéchot J, Lebray P, et al. Direct and indirect evidence for the reversibility of cirrhosis. Hum Pathol. 2006;37: 1519–1526. doi: 10.1016/j.humpath.2006.07.007 16997354

[19] Dufour JF, DeLellis R, Kaplan MM. Reversibility of hepatic fibrosis in autoimmune hepatitis. Ann Intern Med. 1997;127: 981–985. doi: 10.7326/0003-4819-127-11-199712010-00006 9412303

[20] Czaja AJ, Carpenter HA. Decreased fibrosis during corticosteroid therapy of autoimmune hepatitis. J Hepatol. 2004;40: 646–652. doi: 10.1016/j.jhep.2004.01.009 15030981

[21] Falize L, Guillygomarc’h A, Perrin M, Lainé F, Guyader D, Brissot P, et al. Reversibility of hepatic fibrosis in treated genetic hemochromatosis: a study of 36 cases. Hepatology. 2006;44: 472–477. doi: 10.1002/hep.21260 16871557

[22] Vilar-Gomez E, Martinez-Perez Y, Calzadilla-Bertot L, Torres-Gonzalez A, Gra-Oramas B, Gonzalez-Fabian L, et al. Weight Loss Through Lifestyle Modification Significantly Reduces Features of Nonalcoholic Steatohepatitis. Gastroenterology. 2015;149: 367–378 doi: 10.1053/j.gastro.2015.04.005 25865049

[23] Friedman SL, Ratziu V, Harrison SA, Abdelmalek MF, Aithal GP, Caballeria J, et al. A randomized, placebo-controlled trial of cenicriviroc for treatment of nonalcoholic steatohepatitis with fibrosis. Hepatology. 2018;67: 1754–1767. doi: 10.1002/hep.29477 28833331 PMC5947654

[24] Bardou-Jacquet E, Morandeau E, Anderson GJ, Ramm GA, Ramm LE, Morcet J, et al. Regression of Fibrosis Stage With Treatment Reduces Long-Term Risk of Liver Cancer in Patients With Hemochromatosis Caused by Mutation in HFE. Clin Gastroenterol Hepatol. 2020;18: 1851–1857. doi: 10.1016/j.cgh.2019.10.010 31622736

[25] D’Ambrosio R, Aghemo A, Rumi MG, Degasperi E, Sangiovanni A, Maggioni M, et al. Persistence of hepatocellular carcinoma risk in hepatitis C patients with a response to IFN and cirrhosis regression. Liver Int. 2018;38: 1459–1467. doi: 10.1111/liv.13707 29377616

[26] D’Ambrosio R, Colombo M. Should surveillance for liver cancer be modified in hepatitis C patients after treatment-related cirrhosis regression? Liver Int. 2016;36: 783–790. doi: 10.1111/liv.13106 26936383

[27] Semmler G, Meyer EL, Kozbial K, Schwabl P, Hametner-Schreil S, Zanetto A, et al. HCC risk stratification after cure of hepatitis C in patients with compensated advanced chronic liver disease. J Hepatol. 2022; Available from: https://www.journal-of-hepatology.eu/article/S0168-8278(21)02234-0/fulltext. 34871626 10.1016/j.jhep.2021.11.025

[28] DeLeve LD. Liver sinusoidal endothelial cells in hepatic fibrosis. Hepatology. 2015;61: 1740–1746. doi: 10.1002/hep.27376 25131509 PMC4333127

[29] Guido M, Sarcognato S, Russo FP, Cardin R, Piciocchi M, Colloredo G, et al. Focus on histological abnormalities of intrahepatic vasculature in chronic viral hepatitis. Liver Int. 2018;38: 1770–1776. doi: 10.1111/liv.13718 29427537

[30] Couvelard A, Scoazec JY, Feldmann G. Expression of cell-cell and cell-matrix adhesion proteins by sinusoidal endothelial cells in the normal and cirrhotic human liver. Am J Pathol. 1993;143: 738–752. 8362973 PMC1887198

[31] Cui S, Hano H, Sakata A, Harada T, Liu T, Takai S, et al. Enhanced CD34 expression of sinusoid-like vascular endothelial cells in hepatocellular carcinoma. Pathol Int. 1996;46: 751–756. doi: 10.1111/j.1440-1827.1996.tb03544.x 8916144

[32] Kim SU, Oh HJ, Wanless IR, Lee S, Han K-H, Park YN. The Laennec staging system for histological sub-classification of cirrhosis is useful for stratification of prognosis in patients with liver cirrhosis. J Hepatol. 2012;57: 556–563. doi: 10.1016/j.jhep.2012.04.029 22617153

[33] Khan R, Velpari S, Koppe S. All Patients With Advanced Fibrosis Should Continue to Be Screened for Hepatocellular Carcinoma After Sustained Virological Response of Hepatitis C Virus. Clin Liver Dis. 2018;12: 137–139. doi: 10.1002/cld.707 30988930 PMC6385927

[34] Sun Y, Zhou J, Wang L, Wu X, Chen Y, Piao H, et al. New classification of liver biopsy assessment for fibrosis in chronic hepatitis B patients before and after treatment. Hepatology. 2017;65: 1438–1450. doi: 10.1002/hep.29009 28027574

